# Utilizing Contrast-Enhanced Ultrasonography with Phosphatidylserine Microbubbles to Detect Placental Inflammation in Rhesus Macaques

**DOI:** 10.3390/molecules28072894

**Published:** 2023-03-23

**Authors:** Rachel C. Wilson, Jamie O. Lo, Gabriel Romero Jimenez, Jonathan R. Lindner, Ov D. Slayden, Victoria H. J. Roberts

**Affiliations:** 1 Division of Reproductive and Developmental Sciences, Oregon National Primate Research Center, Beaverton, OR 97006, USA; 2 Department of Obstetrics and Gynecology, Oregon Health and Science University, Portland, OR 97239, USA; 3 Cardiovascular Division, University of Virginia Medical Center, Charlottesville, VA 22903, USA

**Keywords:** pregnancy, in vivo imaging, contrast agent, intervillous space, syncytiotrophoblast, selectin p, angiopoietins

## Abstract

The ability to comprehensively monitor physiological and detect pathophysiologic processes early during pregnancy can reduce maternal and fetal morbidity and mortality. Contrast-enhanced ultrasound (CEUS) is a non-invasive imaging technology that utilizes the acoustic detection of microbubbles to examine vascular spaces. Furthermore, microbubbles conjugated to specific compounds can focus studies on precise physiological pathways. We hypothesized that CEUS with phosphatidylserine microbubbles (MB-PS) could be employed to monitor placental inflammation. We tested this hypothesis in rhesus macaques (*Macaca mulatta*), a translational and relevant animal model of human placental health. As placental inflammation impacts many at-risk pregnancies, we performed CEUS with MB-PS in pregnant macaques fed a high-fat diet (e.g., a western-style diet, WSD) in the presence or absence of testosterone (T) to mimic the increased risk of polycystic ovary syndrome and subfertility. We have previously demonstrated a placental inflammation phenotype in this model, and, thus, we related the MB-PS CEUS signal intensity to placental inflammation markers: selectin p and angiopoietins. Testosterone exposure increased the MB-PS signal in the placental microcirculation on the maternal side compared to control animals. We found that T increased placental weight and decreased angiopoietin 2 (ANGPT2) immunoreactivity. Furthermore, a significant inverse correlation was found between MB-PS signal and ANGPT2. This indicated that CEUS with MB-PS can be used to monitor placental parameters. We propose that CEUS with MB-PS could aid in the identification of pregnancies at risk of placental vascular compromise.

## 1. Introduction

A healthy, full-term pregnancy is predicated upon numerous interacting factors, and the proper development and functioning of the placenta is arguably the most important. The principal function of the placenta is to facilitate the maternal and fetal exchange of gases and nutrients across the chorionic villi. The syncytiotrophoblast is the outer most layer of cells on chorionic villi and separates the maternal blood supply from fetal vasculature. Placental inflammation can attenuate the exchange of nutrients and gases between the maternal and fetal circulation and can occur as a consequence of an activated innate or acquired immune response [1]. Less than half of the histologically confirmed cases of placental inflammation occur in the presence of microbial infection indicating the activation of the innate immune system [2]. The activation of innate immunity can be mediated through various factors present on the syncytiotrophoblast, such as Toll-like receptors, complement receptor 5aR, CD55, CD59, and monocyte chemoattractant protein [3,4,5], potentially resulting in placental inflammation.

The maternal diet during pregnancy can influence inflammation in serum and placental tissues [6,7,8]. The proteins of the p38MAPK inflammatory signaling pathway are lower in the placental villi of pregnant individuals consuming a healthier diet [7]. Studies investigating the influence of diet on pregnancy are challenging to perform in humans with correlative or associative data comprising the body of literature. Furthermore, confounding variables are often not addressed in descriptive clinical studies. Accordingly, a translational model is necessary to determine the effect of maternal diet on placental inflammation. Rhesus macaques represent an optimal animal model to study human pregnancy due to similarities in uterine and placental anatomy (e.g., spiral artery remodeling and hemochorial type). Typically, human placentas are comprised of one lobe, while rhesus placentas are often bi-lobed. Although bi-lobed or succenturiate placentas in humans can occur, the incidence is approximately from 2 to 3 out of 1000 patients [9]. The two lobes of the bidiscoid rhesus macaque placenta function as one unit; the morphology and function of the lobes do not differ, but the secondary lobe usually produces a proportionally smaller contribution to the overall physiology of the placenta. In a cohort of eighty pregnancies delivered at different gestational ages, we have found that the primary lobe contributes a range of 60–65% toward the total placental weight [10].

The standard clinical methods for antenatal surveillance include (1) physical examination, (2) biomarkers in maternal serum and amniotic fluid, and (3) observation of the gross anatomical structures of the uterus, placenta, and fetus achieved using noninvasive imaging modalities [11]. Both ultrasonography (US) and magnetic resonance imaging (MRI) can detect pregnancy-related anatomical abnormalities. For US and MRI, perfusion imaging algorithms with contrast agents have been used to interrogate the placenta [12,13,14,15], but there is an abundance of caution with any contrast agent use in pregnant individuals. The application of contrast-enhanced US (CEUS) with microbubbles that reside entirely within the vascular space provides a potentially safer option to examine uterine and placental physiology based on the inert components of the microbubbles used during CEUS. Furthermore, gadolinium, a commonly used contrast agent for MRI, has the ability to cross the maternal fetal interface and accumulate in fetal tissues, albeit at low levels [16,17].

A microbubble formulation approved for clinical imaging is composed of a perfluorocarbon gas core stabilized by a phospholipid microbubble shell [18]. Based on their size and viscoelastic properties, these microbubbles oscillate in the acoustic field when imaged using traditional ultrasound frequencies [18]. Imaging the transit of microbubbles within the microcirculation has been used to quantify tissue perfusion in organs such as the heart, kidney, liver, and placenta [18,19,20,21,22,23,24]. Microbubbles can also be targeted to specific disease-related molecules, particularly those on the endothelial surface, thereby providing a non-invasive method for evaluating vascular phenotypes by detecting retained microbubbles. One form of microbubble targeting has been to incorporate phosphatidylserine (PS) in the microbubble shell (MB-PS) [25,26,27]. The presence of PS in the shell promotes microbubble attachment to activated leukocytes through opsonization, and even adhesion to activated endothelium through mechanisms yet to be fully determined. Imaging MB-PS retention has been used to highlight the inflammation caused by myocardial or renal ischemia-reperfusion injury, angiogenesis, and myocarditis in humans and mice using ultrasound [28,29,30,31]. Ultimately, we are interested in utilizing CEUS molecular imaging with MB-PS as an antenatal surveillance tool for placental dysfunction.

Here, were present proof-of-concept data to suggest that we can extend the application of MB-PS in detecting placenta inflammation. Late in gestation (gestation day (GD) 135), we performed CEUS to collect MB-PS signal data and then performed cesarean sections. We measured the fetal and placental weights to inform the pregnancy outcomes as well as performed immunohistochemistry on the following inflammatory markers in the placental tissue: selectin p and angiopoietin 1 and 2. Selectin p plays a role in the recruitment of leukocytes to sites of injury and inflammation in the cardiovascular system and placenta [32]. Angiopoietins are also present in the placenta and are involved in the inflammatory pathway through mediating angiogenesis and vascular restructuring [33]. The implementation of molecular imaging using contrast-enhanced ultrasound could help to identify at-risk pregnancies, as placental inflammation is associated with placental dysfunction and poor pregnancy outcomes [34,35,36,37].

## 2. Results

### 2.1. In Vivo Imaging with MB-PS

**Figure 1:**
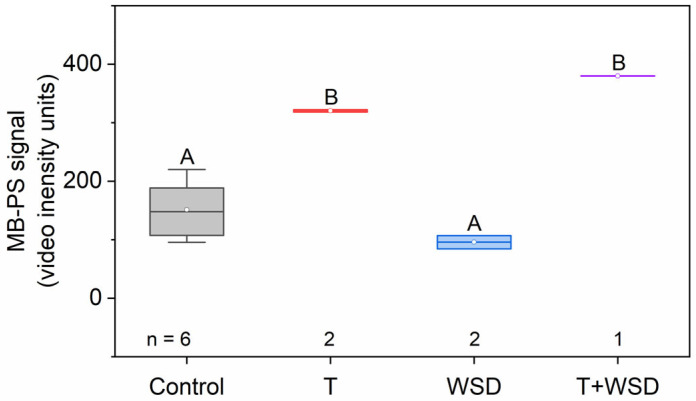
Treatment with testosterone (increases phosphatidylserine-shelled microbubble (MB-PS) signal). MB-PS signal was measured once during pregnancy at gestational day (GD) 135 in female macaques that were fed a control or an obesogenic, western-style diet (WSD) in the presence and absence of subcutaneous testosterone (T) implants. Letters above box plots indicate a significant main effect of T (*t* = 5.91, *p* < 0.001).

We found no significant effect of diet treatment on the MB-PS signal. We found a significant main effect of T on the MB-PS signal with T increasing the MB-PS signal (Figure 1; *t* = 5.91, *p* < 0.001).

### 2.2. Fetal and Placental Weights

**Figure 2:**
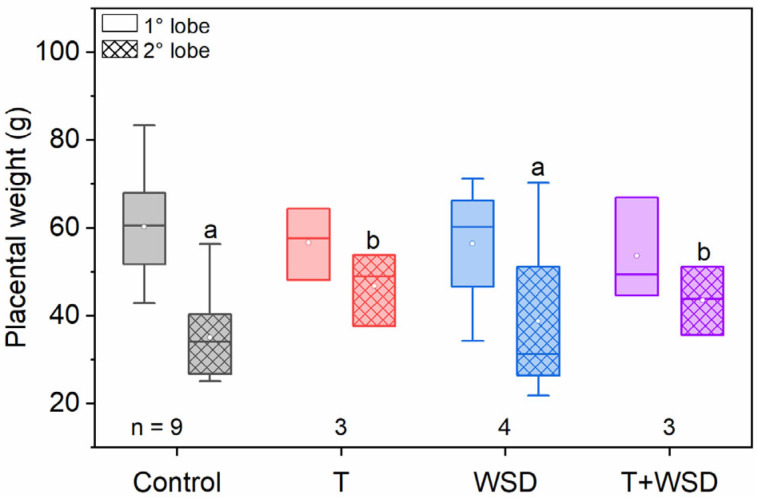
Testosterone (T) increased weights of the secondary (2°) placental lobe. Cesarean section delivery was performed at gestation day 135 after ultrasound procedures. Lettering depicts significant differences for the interaction between placental lobe and T treatment (*F*_1,14_ = 5.11, *p* = 0.04).

The fetal weight did not differ with diet or T but did positively correlate with maternal weight as expected (*F*_1,15_ = 11.17, *p* = 0.004). In addition to finding a significant and positive relationship between maternal and placental weights (*F*_1,14_ = 10.99, *p* = 0.005), we found a significant interaction between the placental lobe and T (Figure 2; *F*_1,14_ = 5.11, *p* = 0.04). While weights of the primary lobe of the placenta were similar across groups, the secondary lobe of the macaques treated with T weighed significantly more than untreated macaques (Figure 2). This finding was unexpected as it appears that T affects placental weight in a lobe-specific manner. Of note, both groups with T treatment had *n* = 3 animals. It remains to be determined whether this outcome will be reproduced in a larger T-treated cohort.

### 2.3. Immunofluorescence for MB-PS and Immunohistochemistry for Inflammation Markers

**Figure 3:**
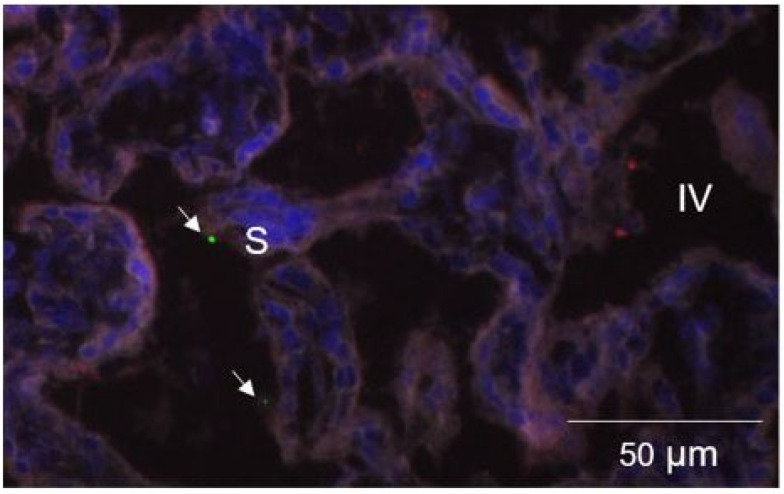
Immunofluorescence of phosphatidylserine-shelled microbubbles (MB-PS). White arrows indicate MB-PS ranging from 1 to 3 μm. MB-PS were only observed in the intervillous space (IV) and associate with the syncytiotrophoblast. Selectin p positive-stained cells in red are also observed but do not localize with MB-PS. Nuclei shown in blue were counterstained with Hoescht.

**Figure 4:**
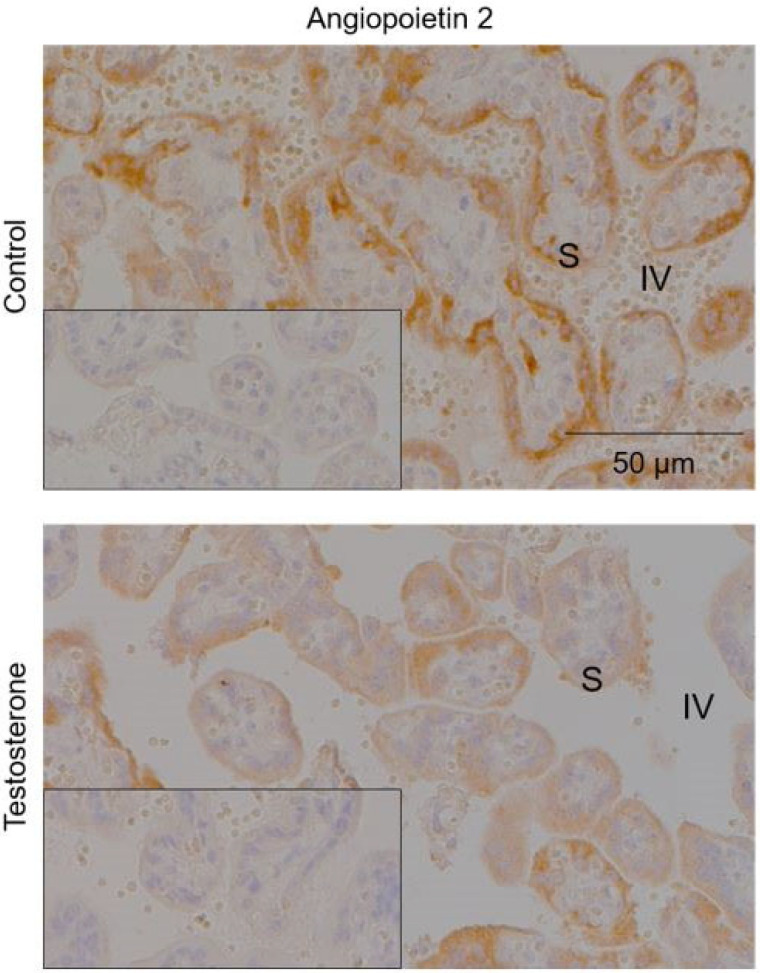
Testosterone attenuated staining of angiopoietin 2 in the syncytiotrophoblast of the placenta. Representative images showing that testosterone significantly decreased angiopoietin 2 staining (brown) in chronic villi (*F*_1,7_ = 5.96, *p* = 0.045). The average ± standard deviation area of staining corrected for total area analyzed was 0.08 ± 0.01 μm for control animals and 0.06 ± 0.01 μm for T-treated animals. Sections are counterstained with hematoxylin (blue). Insets show corresponding negative control sections that were stained using IgG. S: syncytiotrophoblast; IV: intervillous space.

The histologically fluorescently labeled MB-PS were isolated to the intervillous space and ranged from approximately 1 to 3 μm (Figure 3). The microbubbles were not free floating but the MB-PS were associated with the syncytiotrophoblast (Figure 3). The testosterone treatment did not affect selectin p or ANGPT1 but decreased ANGPT2 (Figure 4; *F*_1,7_ = 5.96, *p* = 0.045). On average, the staining area for the control animals was 0.08 ± 0.01 (±standard deviation) μm. The treatment with T decreased the average ANGPT2 staining to 0.06 ± 0.01 μm. We found no effect of WSD on selectin p, ANGPT1, or ANGPT2.

**Figure 5:**
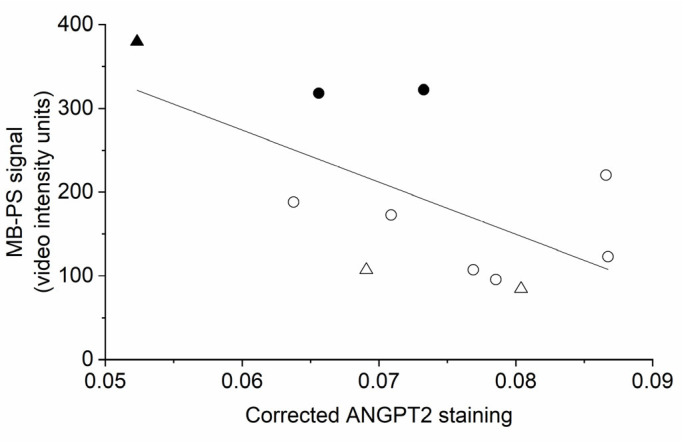
Phosphatidylserine-shelled microbubble (MB-PS) signal and angiopoietin 2 (ANGPT2) display a significant and negative linear relationship (*R*^2^ = 0.31, *p* = 0.045). Circles indicate treatment with a chow, control diet, and triangles a western-style diet. Filled symbols represent treatment with testosterone.

In examining the degree of correlation between the MB-PS signal and inflammatory markers, the linear regression analysis revealed that the MB-PS signal and ANGPT2 were significantly and negatively related (Figure 5; *R*^2^ = 0.31, *p* = 0.045).

## 3. Discussion

We were able to implement an in vivo imaging technology using MB-PS with CEUS to identify impaired placental function in a relevant translational animal model. As proof of principle, the MB-PS signal was elevated in the presence of parameters that suggest placental abnormalities such as increased placental weight and decreased ANGPT2 levels. Furthermore, the MB-PS signal was consistent across groups when no placental abnormalities were detected. Although not currently implemented in pregnancy, because US is relatively inexpensive and widely available, we suggest the potential use of CEUS as an additional diagnostic tool for the clinical monitoring of placental function. The microbubbles do not interfere with hemodynamics, are renally excreted, and, to-date, the safety data do not indicate placental tissue damage following CEUS exposure [14,20,38].

Our findings indicate that endocrinology alterations induce changes in placental anatomy and physiology. It is unlikely that alterations in the uterine artery volume flow influenced the MB-PS signal in pregnant macaques exposed to T as this treatment did not affect the uterine artery flow [21,39]. We demonstrated that treatment with T increased the MB-PS signal and placental weights late at GD 135. Histologically, we showed, for the first time, that MB-PS are associated with the syncytiotrophoblast, which comprised epithelial cells. It is unlikely that the MB-PS were attached to activated leukocytes associated with chorionic villi because we did not observe any nuclear staining near the MB-PS on the intervillous surface of the syncytiotrophoblast. To date, reports only detailed the associations of MB-PS with endothelial cells and leukocytes in the cardiovascular system [26,28,31,40,41,42,43].

As evidenced by alterations in the placenta weight, increased MB-PS signal intensity, and decreased ANGPT2 immunostaining, we provide evidence that T disrupts typical placental physiology. The negative relationship between the MB-PS signal and ANGPT2 staining, and the indirect relationship of T with an increased MB-PS signal and placental weights suggests that utilizing CEUS with MB-PS can assess in vivo physiological parameters. Interestingly, T increased placental weights. Larger placentas are reported to be less efficient [44,45,46]; thus, T alters the ability to transport nutrients into the fetal blood supply. We provide further evidence that T affects cardiovascular parameters in the placenta with our findings that T decreased the immunostaining of ANGPT2 but exerted no effect on ANGPT1. With a higher ratio of ANGPT1 to ANGPT2, vascular stabilization is promoted; whereas, when ANGPT2 is elevated, vascular restructuring occurs [33,47]. Treatment with T also increased the villous volume and decreased the fetal capillary volume in prior pregnancies of this experimental model [39]. Increased villous volumes, decreased fetal capillary volumes, and diminished ANGPT2 levels are all capable of contributing to larger placental weights. An increased villous volume would directly increase the placental weights, though placentas may increase in size in response to lower fetal capillary volumes as a compensatory mechanism. Thus, fetal weights did not significantly differ with T treatment.

The diminished levels of ANGPT2 may also indicate an at-risk pregnancy as ANGPT2 mRNA has been demonstrated to be reduced in pregnant individuals with pre-eclampsia [33], which is a pathophysiological condition of pregnancy featuring both maternal and fetal morbidities. We may be able to identify at-risk pregnancies before the manifestation of clinical diagnostic features (e.g., hypertension, transaminitis, and renal dysfunction) using CEUS with MB-PS. We found that the MB-PS signal was similar in the control and WSD groups when no placental abnormalities were detected. It is difficult to compare with the T+WSD group since the sample size for imaging was one individual. Further investigation earlier in gestation with a larger cohort of animals and validation in humans is necessary to determine if this in vivo imaging procedure can identify the potentially disrupted physiological processes during pregnancy, but, still, it demonstrates early promise.

Our observation of T increasing inflammation in pregnant female macaques was unexpected as evidence in humans and mice, thus suggesting that T exerts anti-inflammatory actions [48,49]. Testosterone primarily acts as a transcription factor through translocation to the nucleus after T binds to its receptor. Immune cells are known to express androgen receptors [48,49]. The evidence to suggest that T is an anti-inflammatory agent primarily emanates from studies performed on male or male-derived cell lines. However, one in vitro study performed on female-derived human monocytes suggests that T can be pro-inflammatory by increasing levels of interleukin-12 and -1β producing monocytes after stimulation with lipopolysaccharide [50]. It is also possible that the metabolism of T to other androgens (e.g., androstenedione and dihydrotestosterone (DHT)) may be responsible for the increase in inflammation we observed. Treatment with DHT decreased the midgestational mRNA levels of ANGPT2 in a pregnant rat model [51]. Although we did not measure DHT in this cohort, treatment with T increases the serum DHT levels in pregnant rhesus macaques [52]. Therefore, the diminished ANGPT2 levels we observed in the T-treated pregnant macaques could be explained by the conversion of T to DHT.

## 4. Materials and Methods

### 4.1. Animal Ethics and Care

The procedures involving animals were approved by the Oregon National Primate Research Center (ONPRC) and Oregon Health and Science University (OHSU) Institutional Animal Care and Use Committee (Approval Code IP0305, Date: 3 January 2019) following the U.S. Public Health Service Policy on Humane Care and Use of Laboratory Animals [53]. The animal care was overseen by the ONPRC Animal Resources and Research Support Unit.

### 4.2. Animal Model

We utilized rhesus macaques (*Macaca mulatta*) exposed to long-term treatment with either a control chow diet or an iso-caloric, fat rich, western-style diet (WSD) in the presence or absence of a subcutaneous implant filled with either cholesterol or testosterone (T) to emulate polycystic ovary syndrome (PCOS). The details of the animal treatments were previously published [54,55,56,57,58]. Briefly, the dietary and T treatments started near menarche and continued for 7 years, at which time the ultrasound procedures were performed. We monitored the serum T levels and replaced implants when the levels dropped below 1 ng/mL. Subfertility is associated with PCOS in people and rhesus macaques [55,59]. Therefore, we examined how these characteristics of PCOS influence pregnancy. Treatment with a WSD and/or T induces obesity and insulin resistance in these animals before and during pregnancy [56,57,58]. Because obesity and insulin resistance are associated with an increase in inflammation [60,61,62], we examined placentas with varying levels of inflammation to evaluate in vivo imaging using MB-PS. The number of pregnancies per group were: Control: *n* = 9, T: *n* = 3, WSD: *n* = 5, and T+WSD: *n* = 3. Due to the availability of custom-prepared MB-PS microbubbles, a subset of these individuals was exposed to the MB-PS procedure once late in gestation: Control: *n* = 6, T: *n* = 2, WSD: *n* = 2, and T+WSD: *n* = 1.

### 4.3. MB-PS Synthesis

Following Mott et al. [26], lipid-shelled MB-PS were prepared by performing a sonication of a decafluorobutane gas-saturated aqueous suspension of 2 mg/mL distearoylphosphatidylcholine, 0.3 mg/mL distearoyl phosphatidylserine (Avanti Polar Lipids, Alabaster, AL, USA), and 1 mg/mL polyoxyethylene-40-stearate (Sigma-Aldrich, St. Louis, MO, USA). The fluorescent lipid-shelled MB-PS were prepared by adding 0.2 mg/mL tetramethylindocarbocyanine perchlorate (Sigma-Aldrich, St. Louis, MO, USA) or 0.2 mg/mL dioctadecyloxacarbocyanine perchlorate (Invitrogen, Waltham, MA, USA) to the gas-saturated aqueous suspension described above.

### 4.4. In Vivo Imaging and Tissue Collection

**Figure 6:**
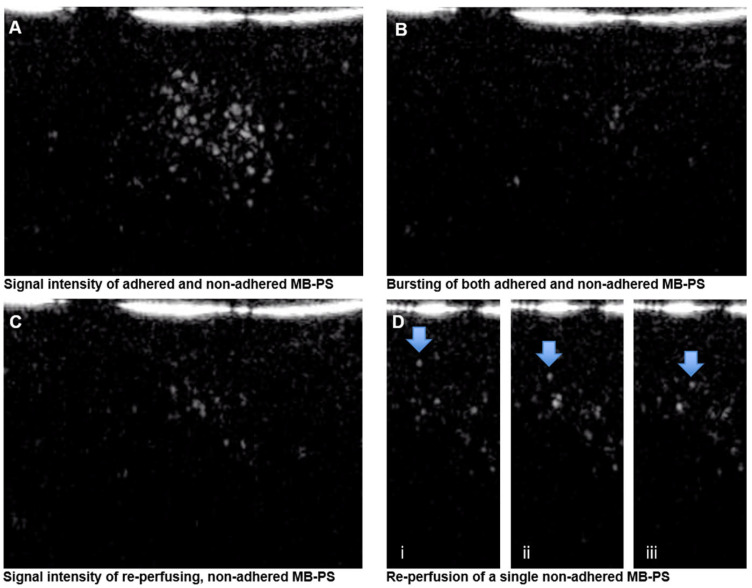
Acquisition of phosphatidylserine-shelled microbubble (MB-PS) signal using contrast-enhanced ultrasonography (CEUS). (**A**) Signal intensity of both adhered and non-adhered MB-PS. (**B**) Bursting of all MB-PS in the frame of view using a high-frequency beam from the ultrasound probe. (**C**) Reperfusion of non-adhered MB-PS. (**D**) Reperfusion of a single MB-PS (arrows) into the intervillous space after bursting of MB-PS across three sequential frames (i–iii). We calculated the MB-PS signal intensity of the adhered bubbles by subtracting panel (**C**) from panel (**A**) to only provide the signal of adhered MB-PS.

The animals (*n* = 11) were imaged on gestational day (GD) 135 where the term is GD 165–168 in the rhesus macaques. For all scans, the pregnant females were initially sedated with intramuscular injections of ketamine (10 mg/kg) and then intubated for continued anesthetization with 1–2% inhaled isoflurane. A venous catheter was placed for maternal administration of the contrast agent. A 1 mL bolus of 10^5^ MB-PS per nL was injected and five minutes was allowed between injection and CEUS imaging to allow for the MB-PS to adhere and also to permit some clearance of non-adhered MB-PS from the circulation [28]. Abdominal contrast-enhanced ultrasonography was performed with a multiphase amplitude-modulation and phase-inversion algorithm on an Acuson Sequoia system (Siemens Medical Systems, Mountain View, CA, USA) that was equipped with a 15L8 transducer at a transmit frequency of 7 MHz with a 0.18 mechanical index. The imaging acquisition was performed with the transducer always maintained at an angle of less than 60° of the vessel. The individual placental cotyledons were imaged by moving the transducer across the maternal abdomen to acquire video recordings from multiple areas of the primary and secondary placental lobes. All in vivo imaging was recorded for data analysis after the procedure. The procedure involves obtaining an initial frame of the placenta that included both adhered and non-adhered MB-PS (Figure 6A). At the start of each imaging study, the gain was set and remained unchanged for all subsequent data acquisition. A high-frequency ultrasound beam with a mechanical index of 1.9 was used to burst all the MB-PS in the field of view (Figure 6B). The burst duration was 5 frames. The focal depth is adjusted for each acquisition to ensure that the ultrasound beam is centered on the region of interest within the placental tissue. The reperfusion of MB-PS back into the intervillous space was then observed (Figure 6C,D). At the end of each clip, the cadence was switched off and B-mode imaging was used to visualize the uterine structures and provide confirmation of the placental outline, which was used to delineate each region of interest for data analysis.

The data was analyzed using the narnar^®^ app (narnar, LLC, Lake Oswego, OR, USA). We were interested in obtaining the signal from only those MB-PS that were adhered to placental tissues as only adhered MB-PS are an indication of activated cells in the cardiovascular space; the activation that likely occurred due to inflammation was induced by experimental treatment. Because the initial ultrasound frame included the signal from both non-adhered and adhered MB-PS, we needed to account for the signal from non-adhered MB-PS. As it is not possible to determine which MB-PS were adhered or not adhered in the initial frame (Figure 6A), we used the signal intensity of reperfusing MB-PS into the intervillous space as a proxy for the signal intensity exerted from the non-adhered MB-PS (Figure 6C,D). We then subtracted the signal from the frame in which MB-PS reperfused into the field of view (i.e., non-adhered MB-PS; Figure 6C) from the signal from the initial frame that included both adhered and non-adhered MB-PS (Figure 6A) to obtain the signal from only those MB-PS that were adhered to placental tissues.

After completion of in vivo MB-PS imaging using CEUS on GD135, the animals were transferred to the surgical services unit for delivery. Prior to cesarean section, a second bolus of fluorescently labeled contrast agent was injected into the venous catheter and MB-PS were allowed to adhere over an 8–10 min incubation period. Ultrasonography was not performed at this time. Instead, the fetus was then delivered and the placenta collected, and the umbilical cord and fetal membranes were trimmed and weighed. The full thickness (maternal decidua to fetal membranes) tissue samples from the placenta were obtained for embedding in a Tissue Tek OCT compound (Sakura Finetek, Torrance, CA, USA) and frozen for cryosectioning, or fixed in 10% zinc formalin and embedded in paraffin.

### 4.5. Immunofluorescence for MB-PS and Immunohistochemistry for Inflammation Markers

Six micron sections of OCT and paraffin-embedded placenta samples were placed on separate SuperFrost Plus slides (ThermoFisher Scientific, Cat# 12-550-15, ThermoFisher Scientific, Waltham, MA, USA). We performed immunofluorescence on the OCT-embedded placenta tissue that was fixed for 2 min in paraformaldehyde to determine if inflammation markers co-localized with the fluorescently labeled MB-PS. The tissue was blocked in 10% donkey serum and then incubated in primary antibody overnight at 4 °C (Selectin p: 1:100 dilution, Abcam, Cat# ab6632, Cambridge, UK). The sections were washed with 0.05 M phosphate-buffered saline (PBS), again blocked in serum, and incubated in donkey anti-mouse secondary antibody conjugated to fluorophore (dilution 1:200, Invitrogen, Alexa Fluor 647, Cat# A31571, Waltham, MA, USA). The sections were counterstained with Hoechst (dilution 1:12, Tocris Bioscience, Cat# 5824, Bristol, UK) and then cover slipped after an application of SlowFade mounting solution (ThermoFisher Scientific, Cat# S36936).

We also performed immunohistochemistry assays to relate the MB-PS signal to the following inflammation markers in the paraffin-embedded samples: Selectin p, angiopoietin 1 (ANGPT1), and angiopoietin 2 (ANGPT2). The immunohistochemistry for each antibody was separately run. The sections were deparaffinized in xylenes and then rehydrated in decreasing concentrations of ethanol. The heat-mediated antigen retrieval was performed using citrate buffer (pH 6) for 5 min. The endogenous peroxidases were quenched in 3% H_2_O_2_ with methanol. After washing in PBS, tissue was blocked in 2% horse serum and incubated in a primary antibody overnight at 4 °C (Selectin p: 1:200 dilution, Abcam, Cat# ab6632, Waltham, MA, USA; ANGPT1: 1:800 dilution, Abcam, ab8451; ANGPT2: 1:800 dilution, Abcam, ab56301). We stained a separate, corresponding section from each tissue sample with an IgG, negative control at the same concentration of each associated primary antibody (rabbit polyclonal, Cat#I-1000, Vector, Burlingame, CA, USA; mouse monoclonal, Cat#5415S, Cell Signaling Technology, Danvers, MA, USA). The sections were again washed in PBS and blocked prior to the secondary antibody incubation of the horse anti-mouse (dilution 1:200, Vector, Cat# BA-2000, Burlingame, CA, USA). The signal was amplified using ABC peroxidase (Vector, Cat# PK-7100) and visualized using 3,3′-diaminobenzidine tetrahydrochloride (Sigma-Aldrich, Cat# D5905, St. Louis, MO, USA). The images of each section were acquired and analyzed using the thresholding tool in ImagePro 10 (Media Cybernetics, Rockville, MD, USA) to identify the total area of positive staining. The positive staining was observed in the from brown to orange color spectrum and separate wavelengths in the red, blue, and green spectra were designated to identify the from brown to orange coloration while ensuring any from purple to blue hues from the hematoxylin staining were excluded. We focused on the staining present in the placental villi by using regions of interest to exclude the intervillous space for each section.

### 4.6. Statistics

All the statistics were performed using SPSS (IBM, Armonk, NY, USA). The data were square root or log-transformed if assumptions of normality and/or equal variance were not met. Because only one individual was subjected to the combined effects of a WSD and T, we performed separate *t*-tests with Bonferroni corrections to determine if either diet or T affected the MB-PS signal. Because placental and fetal weights are indicative of a healthy pregnancy [44,45,46], we examined how treatments affected placental and fetal weights. We utilized the analysis of covariance (ANCOVA) to determine if the fetal weights differed by treatment (WSD ± T) and used maternal weight as the covariate. Likewise, we utilized a repeated measures ANCOVA to determine if placental weights differed with treatment. The within-subjects factor was whether the lobe of the placenta was either primary or secondary, and, again, the maternal weight was used as a covariate. All immunostaining data were corrected for the region of interest areas, and the ANOVAs were performed to determine how treatments affected the staining areas. Finally, we performed linear regressions of inflammatory markers on the MB-PS signal to determine if we could use this noninvasive in vivo technique to produce an inflammatory placental signal.

## 5. Conclusions

Regarded together, our data from a model of compromised placental vascularity demonstrate several potential future avenues of investigation. Studies that examine how diet alters metabolism and nutrient transport during pregnancy are warranted especially considering the influence of developmental processes on health and disease [63]. One potential area of focus includes the identification of pathways that can be utilized to compensate for dietary deficiencies or excess and the extent of their plasticity. The CEUS procedure detailed here can monitor some physiological aspects of the placenta, and the MB-PS signal can be utilized as a proxy for additional vascular and/or metabolic factors. Furthermore, this in vivo imaging technique may aid in the early identification of at-risk pregnancies to guide management and the timing of intervention.

## Data Availability

The data presented in this study are available on request from the corresponding author.
